# Accounting for tagging-to-harvest mortality in a Brownie tag-recovery model by incorporating radio-telemetry data

**DOI:** 10.1002/ece3.1025

**Published:** 2014-03-24

**Authors:** Frances E Buderman, Duane R Diefenbach, Mary Jo Casalena, Christopher S Rosenberry, Bret D Wallingford

**Affiliations:** 1 Pennsylvania Cooperative Fish and Wildlife Research Unit, Pennsylvania State UniversityUniversity Park, Pennsylvania, 16802; 2 U.S. Geological Survey Pennsylvania Cooperative Fish and Wildlife Research Unit, Pennsylvania State UniversityUniversity Park, Pennsylvania, 16802; 3 Pennsylvania Game Commission, Bureau of Wildlife Management2001 Elmerton Ave., Harrisburg, Pennsylvania, 17110

**Keywords:** Auxiliary data, Brownie model, harvest rate, hunter behavior, joint model, known-fate, survival rate, tag recovery

## Abstract

The Brownie tag-recovery model is useful for estimating harvest rates but assumes all tagged individuals survive to the first hunting season; otherwise, mortality between time of tagging and the hunting season will cause the Brownie estimator to be negatively biased. Alternatively, fitting animals with radio transmitters can be used to accurately estimate harvest rate but may be more costly. We developed a joint model to estimate harvest and annual survival rates that combines known-fate data from animals fitted with transmitters to estimate the probability of surviving the period from capture to the first hunting season, and data from reward-tagged animals in a Brownie tag-recovery model. We evaluated bias and precision of the joint estimator, and how to optimally allocate effort between animals fitted with radio transmitters and inexpensive ear tags or leg bands. Tagging-to-harvest survival rates from >20 individuals with radio transmitters combined with 50–100 reward tags resulted in an unbiased and precise estimator of harvest rates. In addition, the joint model can test whether transmitters affect an individual's probability of being harvested. We illustrate application of the model using data from wild turkey, *Meleagris gallapavo,* to estimate harvest rates, and data from white-tailed deer, *Odocoileus virginianus,* to evaluate whether the presence of a visible radio transmitter is related to the probability of a deer being harvested. The joint known-fate tag-recovery model eliminates the requirement to capture and mark animals immediately prior to the hunting season to obtain accurate and precise estimates of harvest rate. In addition, the joint model can assess whether marking animals with radio transmitters affects the individual's probability of being harvested, caused by hunter selectivity or changes in a marked animal's behavior.

## Introduction

Individually marking wildlife is a common technique used to obtain data for estimating population parameters, such as survival and harvest rates. In some cases, more than one data type is available for an individual animal, such as recaptures, resightings, telemetry locations, or recoveries. Recent advances in mark-recapture models include joint models, in which a single model incorporates information from multiple data types. Such models can increase the number of estimable parameters, provide greater precision of estimates, and reduce estimator bias (Nasution et al. 2001). Models have been developed for joint analysis of many combinations of data types (Burnham 1993; Lebreton et al. 1995; Barker 1997; Catchpole et al. 1998; Powell et al. 2000; Nasution et al. 2001; Barker et al. 2004; Gopalaswamy et al. 2012). A combined radio-telemetry and tag-recovery model was developed (Pollock et al. 2004) and implemented (Bacheler et al. 2009) to estimate instantaneous harvest and natural mortality rates within a fisheries context. Although Lakhani (1987) used radio-telemetry data to inform age-specific annual survival rates from a program of marking only juveniles of a nongame species, a model that incorporates radio-telemetry into a tag-recovery estimator for terrestrial game species has not been developed.

Radio-telemetry and tag-recovery programs have strengths and weaknesses associated with implementation and data collection that have to be evaluated in light of research or management goals. Monitoring individuals via radio telemetry can provide more detailed information about individual fates, and these data can be used to estimate survival with a Kaplan–Meier known-fate estimator (Kaplan and Meier 1958). For example, year round data on survival, cause of death, dispersal, and habitat relationships can be collected for specific individuals (Kenward 2001). Conversely, it can be costly to monitor a sufficient number of individuals fitted with radio transmitters to detect biologically important differences among groups (Powell et al. 2000; Kenward 2001; Winterstein et al. 2001; Rogers and White 2007). Also, individual fates must be monitored for the duration of the study and cost depends on the number of field technicians, size of the study area, number of marked individuals, and how frequently an individual must be located (Kenward 2001).

In addition, hunters may notice the presence of a radio transmitter before deciding to harvest an individual (Mayer et al. 2002; Jacques et al. 2011); however, it is unclear if detecting the transmitter influences their decision to attempt harvest (Fuller 1990; Etter et al. 2002; Jacques et al. 2011). Some hunters may not harvest individuals with a radio transmitter because they believe that it is illegal (Jacques et al. 2011) or they are benefitting the research program by foregoing harvest (F. E. Buderman, pers. obs.). A reluctance to harvest an animal with a radio transmitter would result in underestimating the harvest rate of the population. However, if hunters select animals with radio transmitters because of the novelty, this would result in overestimating the harvest rate. In addition, there may be concern that the transmitter itself leads to behavior by the marked individual that makes it differentially susceptible to harvest (Caswell et al. 2012).

In comparison with a radio-telemetry study, a study capturing and marking individuals with inexpensive tags (hereafter called “tags”) should have lower costs per animal marked, although additional expenses are incurred when reward tags from marked animals are subsequently reported. Consequently, more animals can be marked in a tagging study for the same total cost as a radio-telemetry study, although information is only available on harvested individuals (Brownie et al. 1985). A problem with the Brownie tag-recovery estimator, however, is the assumption of no mortality between the time of tagging and the first recovery period (e.g., hunting season). For example, white-tailed deer, *Odocoileus virginianus,* are most easily captured in winter, when there are few alternative food sources and individuals congregate (e.g., Verme 1973; Whitlaw et al. 1998; Norton et al. 2012), but not harvested until autumn; mortality during the 6–9 months between tagging and harvest, though minimal, may negatively bias the harvest rate estimator. Diefenbach et al. (2012) successfully used a Brownie model to estimate spring harvest rates of male wild turkeys, *Meleagris gallopavo*, but such a model for estimating fall harvest rates of females would be inappropriate because of the substantial natural mortality that occurs in the 7–9 months following tagging in late winter.

We develop a joint known-fate tag-recovery model that uses radio-telemetry data to estimate tagging-to-harvest survival and tag-recovery data to estimate harvest and annual survival rates. We use computer simulation to investigate the accuracy and precision of harvest rate estimates using this joint estimator as well as how the allocation of radio transmitters and tags affects precision. We then illustrate use of this joint model for two species with different life-history characteristics: (1) female wild turkey with low tagging-to-harvest survival and low harvest rates, and (2) white-tailed deer with high tagging-to-harvest survival and moderate harvest rates. Also, for the white-tailed deer example, we show how the joint model can test whether individuals fitted with a radio transmitter have different harvest rates either because of hunter selection or behavior of the animal.

## Methods

### Kaplan–Meier known-fate estimator

Radio-telemetry data can be used to estimate survival and harvest rates with the staggered entry design of the Kaplan–Meier known-fate estimator (Kaplan and Meier 1958; Pollock et al. 1989). Survival can be estimated for predefined intervals and modeled as a binomial random variable and estimated by maximizing the likelihood of the product of interval likelihoods: 

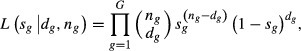
where *s*_*g*_ is survival for each interval *g* (*g *=* *1,…,*G*), *d*_*g*_ is the number of individuals that died in interval *g*, and *n*_*g*_ is the number of animals fitted with radio transmitters that were being monitored at the beginning of interval *g*. The maximum likelihood estimate of an interval survival rate is 

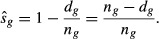


The product of survival rates from consecutive intervals is the cumulative survival rate: 

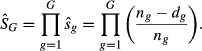


The staggered entry Kaplan–Meier estimator allows individuals to enter and leave the population throughout the study and assumes the following: (1) the sample is representative of the population, (2) survival of individuals is independent of tagging, (3) survival of each individual is independent of other individuals, (4) newly tagged individuals have the same survivorship as previously tagged individuals, (5) censoring, or removing the individual from the population available to be sampled, is random and not related to the fate of the individual (Pollock et al. 1989).

The cost and logistics to affix radio transmitters and monitor a large number of individuals can be prohibitive. For example, the approximate cost of a VHF transmitter for ungulates is $250. A technician can reasonably monitor 40–50 deer with transmitters on a weekly basis, and each full-time technician may cost $20,000/year (C. S. Rosenberry, PGC, pers. comm.). Deer fitted with Global Positioning System (GPS) transmitters do not need to be monitored by a technician, but cost $2,000–3,000. However, if the GPS transmitters store information, as opposed to transmitting data via satellite or cellular telephone networks (an additional cost), they must be monitored and retrieved. Consequently, cost and logistical constraints often limit sample sizes, which can make it difficult to detect biologically important differences between groups. Powell et al. (2000) suggest ≥25 individuals in each marking group are necessary to make such inferences from a combined mark-recapture and radio-telemetry model. For the survival rate analysis, Pollock et al. (1989) recommend a minimum of 40–50 individuals should have radio transmitters at all times, and additional transmitters should be deployed prior to periods of high expected mortality. Winterstein et al. (2001) similarly suggested that 50 individuals were necessary to obtain a 95% confidence interval ±20% about an annual survival rate of 0.50.

In addition, there is concern that the presence of a radio transmitter may influence a hunter's decision to harvest an animal (Fuller 1990; Etter et al. 2002; Mayer et al. 2002; Jacques et al. 2011) because radio transmitters can be visually detected by humans. The estimator may be biased positively or negatively depending on whether hunters avoid or select animals with radio transmitters for harvest. For white-tailed deer hunters, harvest decisions may vary according to characteristics of the deer, such as antler size and sex, and hunter characteristics, such as age, experience, and geographic region (Jacques et al. 2011; PGC, unpubl. data). A confounding cause for biased harvest rates could be that fitting animals with radio transmitters influences their behavior and makes them more susceptible to harvest (Caswell et al. 2012).

### Brownie tag-recovery estimator

Dead-recovery models are special cases of capture–recapture models in which an individual can only be recaptured once as a dead individual (Nichols 1992). The Brownie tag-recovery model is a parameterization of the tag-recovery probability when recovery is a result of hunter harvest, as described by Brownie et al. (1985), and can be used to estimate harvest mortality and annual survival. An individual, upon being tagged, can survive the year (*S*), be killed by a hunter (*K*), or die from natural causes (1 – *S *− *K*). If it is killed by a hunter, the individual could be retrieved (*c*) or not retrieved (1 − *c*). If retrieved, the tag could be reported (*λ*) or not reported (1 − *λ*). In many studies, the probabilities of being killed, recovered, and reported are not separately estimable. The probability of being killed, recovered, and reported (*Kcλ*) is termed the recovery rate (*f*), and the probability of being killed and recovered (*Kc*) is usually termed the harvest rate (*H*). Consequently, when *λ *= 1, such as when sufficiently large rewards are used to encourage reporting of harvests, *f *= *H* (Nichols et al. 1991; Pollock et al. 2001). We assume throughout the rest of this paper that rewards are used to ensure *λ *= 1 and *f *= *H*.

Maximum-likelihood estimators for survival and harvest rate are based on the number of individuals tagged and the number of tagged individuals reported in subsequent hunting seasons (Brownie et al. 1985). We provide the likelihood for the Brownie model in the next section where we incorporate the known-fate data in a joint model. Tag-recovery models assume the following: (1) the sample is representative of the population, (2) age and sex are correctly determined, (3) no tag loss occurs, (4) survival of individuals is independent of tagging, (5) the year of a tag-recovery is correctly recorded, (6) the fate of each tagged individual is independent of the fate of other tagged individuals, (7) the fate of a tagged individual can be represented as a multinomial random variable, (8) all individuals in a given sex–age class have the same annual survival and recovery rates, and (9) annual survival and recovery rates can vary by year, sex–age class, and by area (Brownie et al. 1985).

The primary advantage of tag-recovery studies is that they may be less expensive than other monitoring methods, especially when the cost of capture is low and a large number of individuals can be captured. Likewise, tag-recovery data may be more cost-effective when the cost of using radio transmitters to monitor fates of animals is high. Using reward tags can increase the reporting rate and improve the precision of estimates (Nichols et al. 1991; Pollock et al. 2001). In addition, tags may be useful when a hunter's decision to harvest an individual is dependent on it being marked or unmarked, and the tags are not visible to hunters prior to harvesting an individual.

One disadvantage to tag-recovery models is that according to the parameterization of the model, all of the individuals that are tagged are considered available to be harvested at the start of the first hunting season after tagging. If mortality occurs between tagging and the first hunting season, the harvest estimator is negatively biased. Consequently, studies using tag-recovery models have attempted to capture animals immediately prior to the hunting season. Another disadvantage of tag-recovery models is that cause-specific mortality cannot be determined, although this is irrelevant if the objective is simply to estimate harvest rates.

### Joint Kaplan–Meier known-fate and Brownie tag-recovery model

Incorporating the Kaplan–Meier known-fate estimator into the structure of a Brownie tag-recovery model relaxes some assumptions of the Brownie model and can improve precision of parameter estimates. Animals fitted with radio transmitters are used to account for mortality between tagging and the first hunting season, such that animals fitted with tags do not have to be captured immediately prior to the hunting season. We illustrate the joint model using a monthly Kaplan–Meier known-fate model and a one-age-class Brownie tag-recovery model for a three-year study, but any study duration for estimating tagging-to-harvest survival can be used. We used the Kaplan–Meier estimator as described in the previous section to estimate the cumulative survival rate between tagging and harvest, 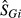, from the monthly survival rates, 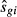, for year *i*, as 

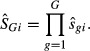


We use the subscript *G* to distinguish this tagging-to-harvest survival rate from the annual survival rate, *S*_*i*_, estimated in the Brownie model.

The standard Brownie tag-recovery likelihood estimates *f*_*i*_ and *S*_*i*_ under the assumption that no mortality occurs between tagging and the first hunting season. However, the additional parameter *S*_*Gi*_ must be estimated if mortality occurs during the tagging-to-harvest interval. The parameters *S*_*Gi*_ and *f*_*i*_ in the following Brownie tag-recovery likelihood are confounded and not separately estimable, and this multinomial likelihood assumes that individuals were only reported in years when individuals also were released: 

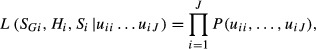




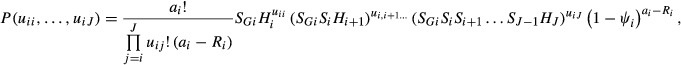


where,

*a*_*i*_ = the number of individuals tagged and released at the start of year *i* (*i *=* *1, …, *J*),

*U*_*ij*_ = the number of individuals tagged in year *i* that are reported in year *j* (*j *= i, …, *J*),

*S*_*Gi*_ = 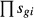 = the tagging-to-harvest survival rate in year *i* (*i *=* *1, …, *J*),

*H*_*i*_ = the harvest rate in year *i* (*i *=* *1, …, *J*),

*S*_*i*_ = the annual survival rate in year *i* (*i *=* *1, …, *J*),


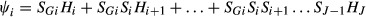
, and


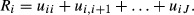


However, the known-fate estimates of survival are independent of the tag-recovery data so the combined likelihood for the joint estimator is simply the product of the two likelihoods: 

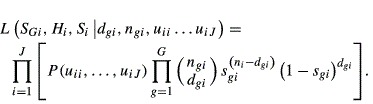


The monthly survival rates estimated with the known-fate data allow *S*_*Gi*_ and *f*_*i*_ to be separately estimable when using the joint Kaplan–Meier known-fate and Brownie tag-recovery estimator (Table 1).

**Table 1:** A three-year, one-age-class joint known-fate tag-recovery matrix showing the expected number of recoveries in year *j* of individuals tagged in year *i* (E[*R*_*ij*_]) based on the number of individuals tagged in year *i* (*N*_*i*_), harvest rate (*H*_*i*_), annual survival rate (*S*_*i*_), and cumulative tagging-to-harvest survival rate (*S*_*Gi*_).

Year	Number released	E[*R*_*ij*_]		
		1	2	3
1	*N*_1_	*N*_1_*S*_*G*1_*H*_1_	*N*_1_*S*_*G*1_*S*_1_*H*_2_	*N*_1_*S*_*G*1_*S*_1_*S*_2_*H*_3_
2	*N*_2_		*N*_2_*S*_*G*2_*H*_2_	*N*_2_*S*_*G*2_*S*_2_*H*_3_
3	*N*_3_			*N*_3_*S*_*G*3_*H*_3_

The joint model can be extended to include data from animals fitted with radio transmitters in the estimation of the harvest rate. If we specify the binomial likelihood of a deer fitted with a radio transmitter being harvested (*f*_*r*_) in year *i* as 

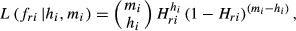
where *h*_*i*_ is the number of animals harvested and *m*_*i*_ is the number of animals available to be harvested, the maximum likelihood estimate of the harvest rate of animals fitted with radio transmitters, for year *i*, is 

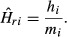


Again, because the harvest rate estimates for animals fitted with radio transmitters are independent of tagged animals, the combined likelihood is simply the product of the individual likelihoods. The likelihood for the joint model can be written as 

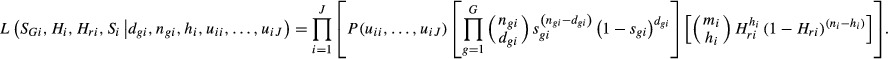


This model can take advantage of additional data from animals fitted with radio transmitters to estimate harvest rates by setting *H*_*i*_ equal to *H*_*ri*_. Additionally, one can evaluate the assumption that animals fitted with radio transmitters have the same probability of being harvested as animals with tags by comparing models with and without *H*_*i*_ = *H*_*ri*_.

### Computer simulations

We evaluated the model for different combinations of radio transmitters and tags using Program SURVIV (White 1983). We performed 1,000 simulations for combinations of numbers of animals fitted with radio transmitters (0, 10, 25, 50, 75, 100, 150, and 200 radio transmitters) and reward tags (50, 100, 200, 300, 400, 500, and 600 tags). We simulated a scenario in which tagging-to-harvest mortality was not accounted for by pairing each reward-tag allocation with 0 radio transmitters and constraining the monthly tagging-to-harvest survival rate to 1.0. We identified two plausible life histories of game species with different tagging-to-harvest survival rates and harvest rates to assess how harvest and annual survival rates affected precision of parameter estimates. Species A had a cumulative tagging-to-harvest survival = 0.90, harvest rate = 0.60, and annual survival rate = 0.30 and species B had a cumulative tagging-to-harvest survival = 0.69, harvest rate = 0.10, and annual survival rate = 0.60. In our simulations, we estimated annual harvest and survival rates even though these parameters were defined as constant, and we did not evaluate models that included age structure, although different harvest and survival rates for each age class can be incorporated into the model.

We evaluated absolute and relative bias, in which the latter was calculated as absolute bias divided by the true value of the parameter, of the standard tag-recovery estimator by averaging the harvest rates over all years across simulations with 0 radio transmitters. We evaluated bias of the joint estimator using averaged harvest rates over all years for simulations with 10, 25, 50, 75, 100, 150, and 200 radio transmitters.

We evaluated the precision of annual harvest rate estimates, 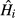, using the coefficient of variation (CV) defined as 

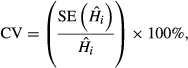
and the coefficient of variation of the root mean squared error (CV(RMSE)), which incorporated both precision and bias, relative to the known value *H*_*t*_: 

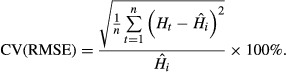


To assist with interpretation, we graphed CV for the harvest rate for each year and species. In addition, we compared CV(RMSE) for each combination of radio transmitters to the scenario in which no radio transmitters were allocated, which would be equivalent to a Brownie tag-recovery estimator.

### Case study: wild turkey

The first case study uses data from female wild turkey where mortality between the winter capture period and the autumn hunting season introduces substantial bias in harvest rate estimates if the joint estimator is not used.

Hen wild turkey were captured in Pennsylvania, January to March, 2010–2012, in which all birds were fitted with an aluminum leg band (1242FR8A rivet band; National Band and Tag, Newport, KY) and a subset of birds were fitted with a Platform Transmitter Terminal (PTT) transmitter (North Star Science and Technology, King George, VA). The PTT transmitters allowed monitoring fates of hens prior to the hunting season (October–November) and estimating survival rates between month of tagging and the hunting season. Leg bands were imprinted with a toll-free number to report the recovery of the band along with wording “$100 REWARD.” We assumed retention of leg bands and reporting rate was 100% (Diefenbach et al. 2000). Because of the January to March capture period, not all turkeys needed to survive the same number of months to the hunting season; individuals tagged in January need to survive 9 months, whereas individuals tagged in March only need to survive 7 months. The joint known-fate tag-recovery model can account for unequal tagging-to-harvest mortality given that not all individuals are tagged at the same time.

We modeled tagging-to-harvest survival by month and age class (adult or juvenile) but pooled data across years because of limited data for the juvenile age class. We modeled tagging-to-harvest survival for each age class because juvenile hens have lower nest-initiation rates and thus should be at lower risk of mortality. However, we combined age classes to model a single harvest rate because by the fall hunting season all tagged birds should have the same risk of being harvested. We developed two joint models, one in which harvest and annual survival rates varied by year (no. parameters = 23), and a second in which harvest and survival rates were constant over time (no. parameters = 20). We developed a third model where the tagging-to-harvest survival rate was 1.0, which is equivalent to a Brownie tag-recovery model that assumes no tagging-to-harvest mortality, to illustrate the effect of assuming no tagging-to-harvest mortality on harvest rate estimates. We used Program SURVIV (White 1983) to maximize the likelihood, obtain parameter estimates and standard errors, and compare models using AIC_*c*_. The wild turkey data and SURVIV code can be accessed from Dryad (doi:10.5061/dryad.26n2q).

### Case study: white-tailed deer

The second case study uses data from white-tailed deer where the survival rate between tagging and the hunting season is >0.90, and we demonstrate how the joint model can be used to assess whether the presence of radio transmitters influences decisions by hunters to harvest deer fitted with radio transmitters.

Pennsylvania has both known-fate and tag-recovery data for adult (≥1-year old) and juvenile (<1-year old) female white-tailed deer for 2009–2011 in Wildlife Management Units (WMU) 2D, 2G, 3C, and 4B. Deer were captured in winter (January–April) when using bait is more effective because of limited food availability, but not harvested until autumn (October–January). Reward tags were small ear tags designed to mimic fur coloration by placing the white stud (6383 blank white stud; Destron Fearing Du™Flex Ear Tags, National Band and Tag Company, Newport, KY) on the inner part of the ear, and the black button retainer (6350 blank black button; Destron Fearing Du™Flex Ear Tags, National Band and Tag Company, Newport, KY) on the outer part of the ear. This design made it difficult for a hunter to identify the deer as tagged prior to harvest. Each tag was labeled with a toll-free telephone number, a unique identification number, and wording indicating a $100 reward (Buderman 2012). To ensure retention of at least one mark until harvest, deer were fitted with a reward tag in each ear. We fitted deer with a VHF radio transmitter (M2510 with PET mortality; Advanced Telemetry Systems, Isanti, MN), GPS transmitter (G2000 Remote Release GPS; Advanced Telemetry Systems, Isanti, MN), or GPS/GSM transmitter (GPS Plus with Global System for Mobile Communications, Vectronic Aerospace, Berlin, Germany). Transmitters were labeled with a toll-free telephone number to report the harvest, but no reward. We monitored survival weekly, and upon the transmission of a mortality signal from a radio transmitter, we investigated the fate of the deer. If a deer with a transmitter was harvested by a hunter and reported via the toll-free number, we recorded date of the harvest mortality.

We first analyzed the known-fate data to identify the best model of tagging-to-harvest survival to simplify the known-fate portion of the model. When estimating monthly tagging-to-harvest survival rates, we considered deer captured at <12 months old to remain juveniles until June, which is the median date of fawn births (PGC, unpubl. data). We pooled data across WMUs because few deer died each month. We developed an *a priori* candidate model set of how harvest and annual survival rates varied among groups. We structured the model to account for deer tagged in January that must survive a longer period of time from tagging-to-harvest than deer tagged in April. We always estimated different harvest rates among WMUs because harvest management decisions were WMU-specific. The candidate set of models evaluated whether harvest rates differed by age class, among years, and between ear-tagged and radio-collared deer. In cases where harvest varied by age or year, annual survival rates also varied by age or year. No annual survival rate is calculated for radio-collared deer, so we did not model a tagging effect for annual survival. We used Program SURVIV (White 1983) to maximize the likelihood, obtain parameter estimates and standard errors, and compare models using AIC_*c*_. The white-tailed deer data and SURVIV code can be accessed from Dryad (doi:10.5061/dryad.26n2q).

## Results

### Simulation results

Absolute bias in the Brownie tag-recovery estimator was less for species A than species B, reflecting the lower harvest rate (Table 2), but relative bias was three times greater for species B compared with species A, corresponding to the lower tagging-to-harvest survival rate. In contrast, the joint estimator was essentially unbiased using both metrics, absolute and relative bias, for species A and B. (Table 2).

**Table 2:** Summary statistics of absolute and relative bias of harvest rates calculated from a standard Brownie tag-recovery model (assuming *S*_*Gi*_ = 1) and a joint known-fate tag-recovery model based on computer simulations for specified tagging-to-harvest survival (*S*_*Gi*_) and harvest (*H*_*i*_) rates.

Model	*S*_*Gi*_	*H*_*i*_	Absolute bias				Relative bias			
			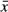	Minimum	Maximum	SD	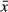	Minimum	Maximum	SD
Species A										
Brownie	0.90	0.600	−0.062	−0.065	−0.060	0.001	−0.104	−0.108	−0.101	0.002
Joint	0.90	0.600	0.002	−0.004	0.010	0.003	0.003	−0.006	0.017	0.005
Species B										
Brownie	0.69	0.100	−0.030	−0.032	−0.026	0.001	−0.303	−0.320	−0.263	0.014
Joint	0.69	0.100	0.002	−0.002	0.013	0.002	0.016	−0.017	0.126	0.024

**Figure 1:**
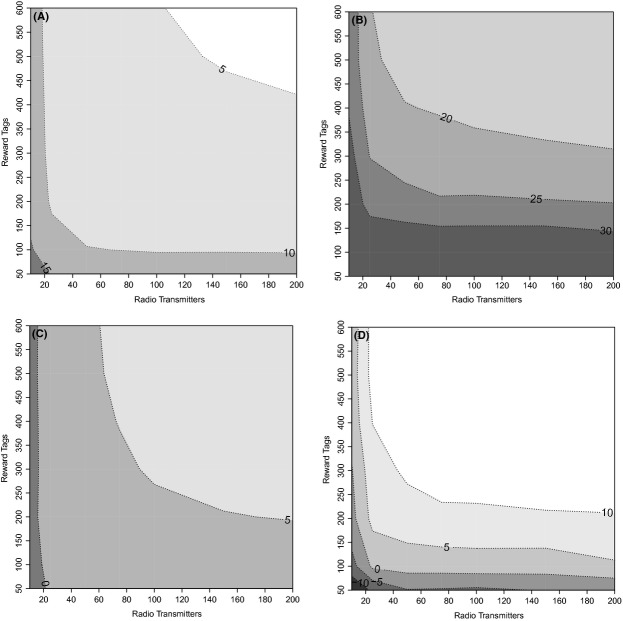
Three-year average CV for harvest rates from a joint known-fate tag-recovery estimator for (A) species A (cumulative tagging-to-harvest survival rate = 0.90, harvest rate = 0.60, annual survival rate = 0.30) and (B) species B (cumulative tagging-to-harvest survival rate = 0.69, harvest rate = 0.10, annual survival rate = 0.60) across radio transmitter allocations from 10 to 200 and three-year average percentage point difference between CV(RMSE) of harvest rates of the Brownie tag-recovery estimator and the joint known-fate tag-recovery model for (C) species A (cumulative tagging-to-harvest survival rate = 0.90, harvest rate = 0.60, annual survival rate = 0.30) and (D) species B (cumulative tagging-to-harvest survival rate = 0.69, harvest rate = 0.10, and annual survival rate = 0.60). Negative values indicate a larger CV(RMSE) for the joint estimator compared with the standard estimator.

As harvest rate decreased across example species, CV across all sample size allocations increased (Fig. 1). For example, precision across all years for species A ranged from 4 to 18% with an average of 8.8% across all simulations. Precision for species B ranged from 15 to 57% with an average of 27.8%, which was approximately three times as high as species A. With only a few radio transmitters to estimate tagging-to-harvest survival, precision of the joint estimator was poor (Fig. 1). This was most likely caused by increased model complexity, because only five parameters would have been estimated in the standard Brownie tag-recovery model, whereas 32 parameters were estimated in the joint model.

For both species, a feasible number of radio transmitters and reward tags would result in a lower CV(RMSE) for the combined model compared with the standard Brownie tag-recovery model. For species A, the joint estimator outperformed the tag-recovery estimator using only 50 reward tags (the minimum tested in this study) and when at least 20 radio transmitters were allocated (Fig. 1). Using 100 reward tags and at least 25 radio transmitters was sufficient for the joint estimator to achieve a lower CV(RMSE) than the tag-recovery estimator for species B (Fig. 1). The difference between CV(RMSE) for the two estimators across the different allocations of reward tags and radio transmitters ranged from −3.22 to 6.61% for species A and from −13.11 to 17.47% for species B, where negative numbers indicate a larger CV(RMSE) for the joint model compared with the Brownie tag-recovery estimator.

### Case study: wild Turkey

We captured and banded 162–169 adults and 74–128 juveniles and fitted 32–55 adults and 6–15 juveniles with radio transmitters per year (Table 3). Survival during the January–September tagging-to-harvest period was 0.452 (SE = 0.0519, 95% CI = 0.35–0.55) for adults and 0.514 (SE = 0.0906, 95% CI = 0.34–0.68) for juveniles. The model with harvest and survival rates varying by year had the lower AIC_*c*_ value (166.3) compared with a constant model (AIC_*c*_ = 169.6). Harvest rates ranged from 0.012–0.045 when we only used data from leg-banded birds and did not account for mortality that occurred between tagging and the hunting season (Table 4). Consequently, not accounting for the tagging-to-harvest survival rate (approximately 50%) would result in harvest rates that were half that of truth. However, the coefficient of variations (CV) for harvest rate estimates for the joint model was similar to the model assuming no tagging-to-harvest mortality (Table 4).

**Table 3:** Number of adult and juvenile hen wild turkeys captured and fitted with $100 reward leg bands or radio transmitters, Pennsylvania, 2010–2012.

Year	Adults		Juveniles	
	Banded	Radio transmitter	Banded	Radio transmitter
2010	162	55	74	6
2011	167	42	128	11
2012	169	32	109	15

**Table 4:** Estimated harvest rates (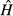) for hen wild turkeys captured as juveniles or adults during January to March in Pennsylvania, 2010–2012, for a joint known-fate tag-recovery model that incorporated mortality between tagging and the first hunting season compared with a model that assumed no tagging-to-harvest mortality.

Year	Incorporating tagging-to-harvest mortality				Assuming no tagging-to-harvest mortality			
	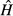	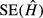	CV[Table-fn tf4-1]	95% CI	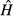	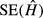	CV^1^	95% CI
2010	0.054	0.024	41.8	0.01–0.10	0.028	0.011	40.2	0.01–0.05
2011	0.088	0.0251	28.5	0.05–0.15	0.045	0.0116	25.8	0.02–0.07
2012	0.022	0.0109	49.5	0.01–0.05	0.012	0.0059	49.2	0.00–0.02

1 CV = 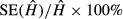.

### Case study: white-tailed deer

Between 0 and 69 deer were available in each month for each sex, age, and WMU group and between 0 and three deer died during each monthly interval. Annual WMU-specific availability of radio-collared females over the hunting season ranged from 10–55 for adults and 0–15 for juveniles, with ≤7 adults and ≤3 juveniles harvested each year in each WMU. If no radio-tagged deer were available during a harvest interval, harvest rate was constrained to equal 0. For the Brownie tag-recovery portion of the analysis, 39–82 adult and 7–50 juveniles were tagged each year in each WMU, with 1–9 adults and ≤9 juveniles harvested each year in each WMU.

The best monthly survival model, obtaining 28% of the support, included only an age effect, with adult monthly survival equal to 0.9905 (SE = 0.0014) and juvenile monthly survival equal to 0.9799 (SE = 0.0048) across individuals from all WMUs. Because little mortality occurred between tagging and harvest, harvest rates from a Brownie model that was equivalent to the best joint model but did not account for tagging-to-harvest mortality were underestimated by only 0.005–0.009. Support among the candidate model set for females was split between a model with no age, tag type, or annual variation (AIC_*c*_ weight = 0.50) and a model with different harvest and survival rates by age class (AIC_*c*_ weight = 0.45; Table 5). No models that included an effect of tag type on harvest had an AIC_*c*_ weight >0.01, such that we were not able to detect any evidence of radio transmitters affecting the behavior of hunters to harvest a deer or deer acting in a way that makes them more likely to be harvested (Table 6).

**Table 5:** Model selection statistics for joint known-fate and tag-recovery models of white-tailed deer harvest and annual survival rates for wildlife management units 2D, 2G, 3C, and 4D, Pennsylvania, 2009–2011. All models separately estimated harvest and survival rates by wildlife management unit. The null model estimated harvest and annual survival rates by wildlife management unit but with no variation among age class, year, or tag type.

Variables	*K*^1^	Log-likelihood	ΔAIC_*c*_	AIC_*c*_ weight
Null	10	−328.7	0.0	0.50
Age class	18	−320.8	0.2	0.45
Year	22	−319.5	5.8	0.03
Tag type	14	−328.2	7.1	0.01
Age and tag type	26	−317.7	10.2	<0.01
Age and year	42	−305.8	18.7	<0.01
Tag type and year	34	−316.4	23.7	<0.01
Age, tag type, year	64	−294.7	41.2	<0.01

Number of parameters.

**Table 6:** Estimated harvest rates (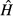) for ear-tagged and radio-tagged female white-tailed deer for the best joint known-fate tag-recovery model that included a tag effect, Pennsylvania, 2009–2011.

WMU	Ear tagged		Radio tagged	
	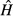	SE( 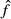 )	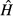	SE( 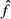 )
2D	0.138	0.0234	0.145	0.0447
2G	0.116	0.0248	0.109	0.0265
3C	0.111	0.0196	0.150	0.0461
4B	0.133	0.0209	0.152	0.0351

## Discussion

To obtain accurate harvest estimates from a Brownie tag-recovery model for study designs where tagging-to-harvest mortality occurs, auxiliary information regarding tagging-to-harvest survival must be available. Otherwise, tagging-to-harvest mortality results in underestimation of the harvest rate. When the tagging-to-harvest survival rate was 0.90, harvest rate was underestimated by about 10% of the true parameter value and when the cumulative tagging-to-harvest survival rate was approximately 0.70, harvest rate was underestimated by 30% of the true value of the estimate. Whether natural resource managers should be concerned about potential bias depends on how underestimating harvest rates will influence management decisions. However, when tagging-to-harvest mortality data are available, they can be used in the joint known-fate tag-recovery model to minimize bias with little loss of precision.

In the computer simulations, harvest data from radio transmitters did not contribute to harvest rate estimation, which is why there was little benefit to deploying more than 20 radio transmitters. If harvest data from radio transmitters were used to inform harvest rates, precision would have improved as more transmitters were deployed. However, whether this would be cost-effective would depend on study objectives and the additional costs of transmitters and personnel. We did not compare the joint model to a model with just radio transmitters, because the assumption of no tag-bias could not be addressed. However, if transmitters are small and not visible, and tagging-to-harvest mortality is minimal, then harvest can be estimated directly from a Kaplan–Meier estimator. However, this may not be a cost-effective strategy if there is a high degree of mortality in the tagging-to-harvest interval. For example, to have a given number of turkeys with transmitters alive at the beginning of the hunting season, twice as many would have to be captured during the trapping season.

A sufficient level of precision (CV) for parameters used to make wildlife management decisions has been described as ≤12.8% (Robson and Regier 1964; Skalski and Millspaugh 2002). However, the required CV may be dependent on the harvest rate of the organism and the management goals. Given a life history similar to species A, one with high harvest rates and low annual survival rates, the desirable CV range of 10–15% was attainable by allocating 10–20 radio transmitters and ≥50 reward tags. Conversely, 47 radio transmitters would have to be deployed to achieve a CV of 12.5% using only a Kaplan–Meier estimator for harvest rates. As harvest rate declines to approximately 0.10, and tagging-to-harvest survival decreases to approximately 0.70, a CV of 10–15% for harvest rate estimates was not achievable given less than 200 transmitters and 600 reward tags. A number of possible sample size allocations, however, from 30 radio transmitters and 600 reward tags to 200 radio transmitters and 350 reward tags could achieve a CV of 20%. In contrast, using only radio transmitters to estimate harvest rate would require 350 radio transmitters to achieve a CV of 20%. We found, across both model species, only about 20 radio transmitters were required for the joint model to achieve levels of precision equivalent to the Brownie tag-recovery model, which assumes no tagging-to-harvest mortality occurred.

In cases in which the joint model is being employed to reduce estimator bias, CV(RMSE) may be a more representative metric than CV for determining when using a joint model would be most beneficial. This was particularly true with the harvest rate estimates for species B, which would have exhibited more bias from tagging-to-harvest mortality than species A. By allocating at least 20 radio transmitters and 100 reward tags the joint model could improve CV(RMSE) by 0–15 percentage points for a species with low tagging-to-harvest survival (≤0.70). In scenarios with high tagging-to-harvest survival, the benefit of incorporating radio transmitters to reduce bias may be negated by the loss of precision. For high harvest rates, 0.60 in the case of species A, the CV(RMSE) of the joint estimator was never more than twice that of the Brownie tag-recovery estimator. However, when the tagging-to-harvest survival rate was low, the reduction in bias was much more likely to warrant incorporating radio transmitters into the study design. Generally, only 20–25 radio transmitters were needed to improve CV(RMSE) of the joint estimator over the Brownie tag-recovery estimator regardless of the tagging-to-harvest survival of the study species.

More complex models may be used to estimate harvest rates with the joint model than the simple model we evaluated via computer simulation (annual variation with no age or sex structure), but we would expect precision to decrease as model complexity increases. For example, including age structure would double the number of parameters (see white-tailed deer case study, Table 5). Another type of complexity that can be modeled with the joint known-fate tag-recovery model is differences in harvest rates for animals fitted with radio transmitters versus those fitted with simple tags. Most studies of harvest rates on wildlife species have monitored individuals with radio transmitters and assume that they are harvested at the same rate as individuals without transmitters. However, there is concern that the presence of a visible transmitter may affect a hunter's decision to harvest an individual (Fuller 1990; Etter et al. 2002; Mayer et al. 2002; Jacques et al. 2011) or that the transmitter leads to behavior by a marked individual that increases the probability it will be harvested (Caswell et al. 2012), leading to harvest estimates that are not representative of the population. Moreover, it is not clear how selective harvest by hunters might influence harvest estimates because hunters could avoid harvesting animals with visible radio transmitters in the belief they are illegal to harvest or that harvest would adversely affect the research project (F. E. Buderman, pers. obs.). Conversely, animals fitted with a radio transmitter could be viewed as a novelty and thus more likely to be harvested.

To date, research to address the potential bias introduced by using radio transmitters to estimate harvest rates have used hunter questionnaires or simulated hunting situations to assess hunter harvest behavior (e.g., Jacques et al. 2011). Such studies may provide insight into hunter behavior but are fraught with the difficulty of assessing whether a hunter's response to a hypothetical situation would be the same as their response to a real harvest opportunity. The model we developed is an opportunity to objectively assess the effect of transmitters on harvest by using animals marked with visible radio transmitters and less visible tags to separately estimate harvest rates and statistically evaluate if parameter estimates differ. However, this method does not allow differentiation between hunters selecting for or against transmitters and marked individuals behaving in a way that makes them more vulnerable to harvest. If it were determined that there was no effect of the visible radio transmitter on harvest rates, as we found with female white-tailed deer in Pennsylvania, animals with radio transmitters can be used to increase the sample size (precision) for harvest rate estimates.
